# Supplementary material for: Bacterial profile of bovine mastitis in Ethiopia: a systematic review and meta-analysis
Source: PeerJ. 2022 May 6;10:e13253. doi: 10.7717/peerj.13253 (PMC9083533; doi:10.7717/peerj.13253)
Supplement: Supplemental Information 1 [file peerj-10-13253-s001.docx]

According to literatures, bovine mastitis is important causes of economic losses in dairy industry in Ethiopia. Over the years, a number of researchers have been reported the prevalence of bovine mastitis in Ethiopia. Accordingly, the apparent prevalence of mastitis falls within the range from 9.12% to 88.9%. As reports it is second ranked and most important cause of milk production loss directly and devastating disease with a higher incidence leading to culling of dairy cows. Despite the numerous reports on the widespread occurrence of mastitis in different parts of the country, a systematic review and pooled quantitative documentation of the status of bacterial mastitis has not established so far. In recent times it is of paramount importance to consolidate the pooled prevalence estimates and develop an action plan to control and manage the disease that would help to reduce its prevalence and effects.

To undertake considerable measure, accurate estimates of pooled prevalence of bovine mastitis giving emphasis on bacterial pathogens are paramount, where the critical dairy mastitis and pathogen burden control programs are if prioritized. We believe that the findings presented in our paper will appeal summarized, pooled, estimate from literatures on a topic to provide a readily available pooled source of information on which to base actions in the practice of evidence-based medicine or to direct policy decisions who subscribe to the PeerJ. In doing so, we hope that our manuscript will be baseline, is used to develop the control programs required to minimize the dairy cattle losses directly and its economic significant effects indirectly.
